# Mobile phones and head tumours. The discrepancies in cause-effect relationships in the epidemiological studies - how do they arise?

**DOI:** 10.1186/1476-069X-10-59

**Published:** 2011-06-17

**Authors:** Angelo G Levis, Nadia Minicuci, Paolo Ricci, Valerio Gennaro, Spiridione Garbisa

**Affiliations:** 1 Department of Experimental Biomedical Sciences, Medical School of Padova, Padova, Italy; 2 Institute of Neuroscience, National Research Council, Padova, Italy; 3 Local Sanitary Unit, Mantova, Italy; 4 National Cancer Research Institute (IST), Genova, Italy

## Abstract

**Background:**

Whether or not there is a relationship between use of mobile phones (analogue and digital cellulars, and cordless) and head tumour risk (brain tumours, acoustic neuromas, and salivary gland tumours) is still a matter of debate; progress requires a critical analysis of the methodological elements necessary for an impartial evaluation of contradictory studies.

**Methods:**

A close examination of the protocols and results from all case-control and cohort studies, pooled- and meta-analyses on head tumour risk for mobile phone users was carried out, and for each study the elements necessary for evaluating its reliability were identified. In addition, new meta-analyses of the literature data were undertaken. These were limited to subjects with mobile phone latency time compatible with the progression of the examined tumours, and with analysis of the laterality of head tumour localisation corresponding to the habitual laterality of mobile phone use.

**Results:**

Blind protocols, free from errors, bias, and financial conditioning factors, give positive results that reveal a cause-effect relationship between long-term mobile phone use or latency and statistically significant increase of ipsilateral head tumour risk, with biological plausibility. Non-blind protocols, which instead are affected by errors, bias, and financial conditioning factors, give negative results with systematic underestimate of such risk. However, also in these studies a statistically significant increase in risk of ipsilateral head tumours is quite common after more than 10 years of mobile phone use or latency. The meta-analyses, our included, examining only data on ipsilateral tumours in subjects using mobile phones since or for at least 10 years, show large and statistically significant increases in risk of ipsilateral brain gliomas and acoustic neuromas.

**Conclusions:**

Our analysis of the literature studies and of the results from meta-analyses of the significant data alone shows an almost doubling of the risk of head tumours induced by long-term mobile phone use or latency.

## Background

The worldwide spread of the use of MPs (mobile phones: analogue and digital cellulars, and cordless) has heightened concerns about possible adverse effects, especially head tumours. According to the International Telecommunications Union, the number of cell-phone subscriptions has reached 5 billion (mid 2010), with more than half of all users believed to be children and young adults. There are no data for cordless users, but a figure of 2 billion is a reasonable assumption. Given these numbers, even an established modest increase (20-30%) in tumour risk for MP users would result in significant social and health costs and individual suffering, while higher risks could give rise to a health crisis of dramatic proportions. While most technologies carry risks, these should be assessed accurately and responsibly.

MPs were introduced onto the market in the 1980s, and widely used for the following decade in the USA, the Scandinavian countries and Israel. Since the beginning of 1990s MPs have become widespread in many other countries too, with the consequence that there has been exposure to MP radiation throughout almost the entire world for at least 20 years [1-3]. Although brain and cranial nerve tumours may have very long latency times (up to 30 years or more), it is likely that - as found with long-latency tumours due to ionizing radiations, asbestos or smoking - some due to MP will be diagnosed after just 10-15 years of MP use or latency.

The case-control studies by the Hardell group in Sweden report a statistically significant increase of at least 100% in risk of ipsilateral cerebral cancers (astrocytomas, a highly invasive glioma sub-type) and of benign tumours of the acoustic nerve (neuromas) among MP users, after use or latency period ≥ 10 years [1-3]. It is therefore vital to understand the weight of the conflicting data from other studies which are considered reassuring in their failure to find any increased risk of head tumours in MP users [4,5].

## Methods

We have carried out a critical examination of the protocols and results from all case-control and cohort studies, pooled analyses and meta-analyses on head tumour risk among MP users. For each study we have identified the elements that must be taken into account to ensure an impartial evaluation of its reliability, that is: a) the number of subjects selected (cases and controls), and the percentage of their participation in the study; b) the percentage of actually exposed subjects, based on the frequency and duration of the MP use; c) the inclusion among the exposed of all users of MPs, cordless included; d) the latency and/or exposure time since first use of MPs; e) the laterality of the head tumour localization relative to the habitual laterality of MP use; f) the distribution of the relative risk (odds ratio, OR) values above and under 1, their statistical significance [95% confidence interval (95% CI) limits], and the probability that such distribution might be casual; g) the full and correct selection and citation of data included in the meta-analyses.

We have quantified the total number of OR values from each study, independently of sex, age, exposure time or latency of the examined subjects. Since the OR estimates reported by each author are not independent, a statistical comparison between the percentages of ORs > 1 or < 1 is difficult. However, a simple comparison of their percentage may indicate if their differences are more or less random, and might be due to a significantly increased risk or a substantial protective effect, or else - in the absence of plausibility of either of these effects - to errors and/or distortions in the study design.

In order to be included in our meta-analyses, studies had to have met all the following criteria:

• published in peer-reviewed journals;

• included participants using MPs since ≥ 10 years;

• incorporated a laterality analysis of tumours.

The hypothesis test for presence of heterogeneity was based on the Q test of heterogeneity, which follows a χ^2 ^distribution. Furthermore, two measures for quantifying the impact of heterogeneity were calculated: H^2 ^(square root of the Q heterogeneity statistic divided by its degrees of freedom) and Higgins I^2 ^(transformation of H that describes the proportion of total variation in study estimates that is due to heterogeneity). If heterogeneity was observed, then the random-effect model was performed by incorporating an estimate of the between-study heterogeneity (DerSimonian and Laid τ^2^) into the weights. When the general fixed effect model was applied to each study estimate, a weight directly proportional to its precision was given (inverse variance-weighted method) [6].

## Results

### MPs and head tumours: positive data

An overview of the most significant results obtained by the Hardell group in the three pooled analyses of their data through case-control epidemiological studies referring to tumours diagnosed during 1997-2003 is given in Table 1[1-3] With ≥ 10-year MP use or latency, a statistically significant (s.s.) increase (ca 2- to 4-fold) in risk of overall (ipsi- plus contralateral) malign and benign brain tumours and acoustic neuromas is shown after use of analogue and digital cellulars. With cordless phone use, instead, risk is about double, s.s. only for malign brain tumours (Table 1).

**Table 1:** Results from the case-control studies by Hardell.

tumours		analogue		digital		cordless	
brain malign		(82/84):	2.4; 1.6-3.4	(19/18):	2.8; 1.4-5.7	(33/45):	1,8; 1,1-3.0
only astrocytomas I-IV		(59/84):	2.7; 1.8-4.2	(15/18):	3.8; 1.8-8.1	(23/45):	2.2; 1.3-3.9
brain benign		(57/84):	1.8; 1.2-2.6	(13/18):	1.6; 0.8-3.5	(28/45):	1.4; 0.8-2.3
only meningiomas		(34/84):	1.6; 1.02-2.5	(8/18):	1.3; 0.5-3.2	(23/45):	1.6; 0.9-2.8
acoustic neuromas		(19/84):	3.1; 1.7-5.7	(1/18):	0.6; 0.1-5.0	(4/45):	1.0; 0.3-2.9
							
Idem, but also as a function of head tumour laterality [3] (≥ 10-year latency)							

**tumours**	**MP type**	**all**		**ipsilateral**		**contralateral**	

astrocytomas	analogue + digital	(78/99):	2.7; 1.8-3.9	(50/45):	3.3; 2.0-5.4	(26/29):	2,8; 1,5-5.1
″	cordless	(28/45):	2.5; 1.4-4.4	(19/15):	5.0; 2.3-11.0	(8/20):	1.4; 0.6-3.5
others malign	analogue + digital	(8/99):	3.2; 1.2-8.8	(4/45):	4.1; 1.03-16.0	(1/29):	1.7; 0.2-15.0
″	cordless	(1/45):	1.1; 0.1-10.0	-	not analysed	(1/20):	3.9; 0.3-44.0
neuromas	analogue + digital	(20/99):	2.9; 1.6-5,5	(13/45):	3.0: 1.4-6.2	(6/29):	2.4; 0.9-6.3
″	cordless	(4/45):	1.3; 0.4-3.8	(3/15):	2.3; 0.6-8.8	(1/20):	0.5; 0.1-4.0
meningiomas	analogue + digital	(38/99):	1.5; 0.98-2.4	(18/45):	1.6; 0.9-2.9	(12/29):	1.6; 0.7-3.3
″	cordless	(23/45):	1.8; 1.01-3.2	(11/15):	3.0; 1.3-7.2	(7/20):	1.1; 0.5-2.9
							
Idem, only individuals who started using MPs < 20-year old (≥ 1-year latency) [3,16]							

**tumours**	**MP type**	**all**		**ipsilateral**		**contralateral**	

astrocytomas	analogue + digital	(15/14):	5.2; 2.2-12.0	(8/5):	7.8; 2.2-28.0	(2/4):	2.2; 0.4-13.0
″	cordless	(14/16):	4.4; 1.9-10.0	(9/6):	7.9; 2.5-25.0	(1/4):	1.1; 0.1-10.0
neuromas	analogue + digital	(5/14):	5.0; 1.5-16.0	(3/5):	6.8; 1.4-34.0	(1/4):	2.4; 0.2-24.0
″	cordless	(1/16):	0.7; 0.1-5.9	(1/6):	1.7; 0.2-16.0	-	not analysed

Results of the *pooled analyses *by Hardell [1-3] on the risk of overall head tumours in exposed subjects compared to that of non-MP-users, as a function of the use of different MP types, (no. of cases and controls with ≥ 10-year use or latency): OR; 95%CI.

95% s.s. data

As Table 1 shows, the tumour increase is chiefly localized on the habitual-use side of the head (ipsilateral tumours), and is very marked (up to 3-5 times normal incidence) and s.s. for malign brain tumours and acoustic neuromas with cellular phone use, and for astrocytomas and meningiomas with cordless use. The data for overall tumours are lower, though still considerable (up to 2-3 times normal incidence) and s.s., while the risk of contralateral tumours is not s.s., except for astrocytoma following use of cellular phones. According to Hardell, this latter finding results from the fact that the radiation produced by MPs - despite being much lower on the contralateral side - is still significant in the ventricular and subventricular space from which gliomas and (their subtype) astrocytomas originate, such that these can develop also to the contralateral side. Finally, the increase in risk of cerebral astrocytomas and acoustic neuromas, in particular ipsilateral, is higher in the subgroup that started using MPs at an age <20 years, even if the 95%CIs are very broad, owing to the still-limited number of subjects being studied (Table 1).

It should be stressed that a greater increase in ipsilateral tumours than in total tumours, but absence of increase in contralateral tumours, is precisely what would be expected in the case of MPs having oncogenic action [4,5].

**Figure 1:**
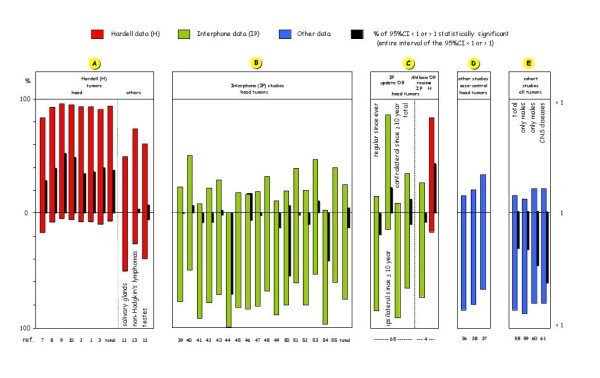
**Hardell and Interphone data: percentage of the OR values > 1 or < 1, and percentage of those statistically significant**.

A detailed analysis of the data from Hardell's seven most recent studies [7-10], including the pooled analyses [1-3], shows that (see additional file 1):

• the percentage participation in the epidemiological study is always very high (84-91%) for both cases and controls;

• the percentages of people exposed are sizeable (mean = 60%, but - in a few studies - up to 70-80%) for both cases and controls;

• MP use is significant: 194 cases used MPs for more than 1000 hours, and 85 for more than 2000 hours, for at least 10 years (i.e. from > 16 to just > 32 min/day);

• the percentages of cases and controls exposed for at least 10 years are 18% and 13% of the total number of exposed cases and controls;

• of the total OR values reported in the above studies, over 90% are > 1, 37% of which are s.s., and the probability of this highly asymmetrical distribution of OR values being due to chance is almost zero (Figure 1A). This pattern indicates that the results are not due to errors or conditioning in the protocol Hardell used, since in other reports regarding other types of tumour (salivary glands [11] and testicles [12]) in MP users - due to the very limited number of those exposed for at least 10 years - no s.s. risk increase is found, nor is there any clear prevalence of OR values > 1. Only for non-Hodgkin lymphomas [13] a s.s. risk increase is found and the distribution of OR values is shifted towards values > 1 (73%), with low probability of this being due to chance (Figure 1A);

• the increased risk in MP users is not limited to gliomas, meningiomas and acoustic neuromas, but involves also other types of head tumour -low grade and high grade astrocytomas, oligodendrogliomas, medulloblastomas, ependimomas, and other/mixed malignant tumours; pituitary adenomas and other/mixed benign brain tumours - which are considered separately [3].

In the Hardell group studies [1-3,7-10,14] a dose/response relationship and thus the existence of a cause-effect relationship are documented by the fact that:

• the risk of developing tumours is prevalent, if not exclusive, on the head side habitually exposed to MP radiation (ipsilateral);

• the trend for increase in OR as a function of time of MP use is s.s.;

• the risk is higher in rural areas [15], where the signal required for optimal use of cellular phones is often very limited owing to the low number of base-stations, and the compensatory emission of the cell-phone battery is particularly high (up to 80 V/m or more) compared with urban areas where the signal is almost always optimal, and the battery emission is a minimum (1 V/m or even less);

• the combined use of various types of MP raises the risk of developing head tumours;

• the risk of head tumour is higher in those starting MP use when aged below 20 years [3,16] (see also Table 1).

The biological plausibility of the oncogenic action of the EM radiation emitted by the MPs is supported by a considerable amount of experimental data [17-19]. This radiation, in fact, can produce a variety of effects able to cause or contribute to the neoplastic cell transformation:

• genetic alterations (DNA damage, chromosomal aberrations, micronuclei, sister-chromatid exchanges and gene mutations) in cells irradiated *in vitro *(including germinal and cerebral cells) [20,21]; in animals exposed in the laboratory [22,23] or natural environment [24], and in MP users [25];

• induction of DNA reparative synthesis and alterations in transcription of DNA, activation of oncogenes and other epigenetic effects [26,27];

• alteration of the blood-brain barrier permeability and brain neuron damage [28,29];

• induction of heat-shock proteins and apoptosis that stress living cells [30,31];

• reduction of melatonin synthesis and activation of Fenton's reaction which increase the concentration of free radicals and peroxides able to damage the DNA [20,32];

• alteration of functionality, count and form of sperms in MP users whose phone stays on and in their trouser pockets during the call [33,34].

It should be noticed that many studies on biological effects of MP use are negative, but for the major part were conditioned having been funded by the cell-phone industry [35] (see Discussion).

### MPs and head tumours: negative data

Between 2000 and 2002 three case-control studies were published - two were funded by MP companies [36,37], while in one no information was given about how the study was funded [38]. The findings indicated no increase in risk of brain or acoustic nerve tumour associated with MP use. However, there was complete absence of subjects exposed for at least 10 years, and the maximum latency period was only 4-5 years (see additional file 2).

On this basis, it is small wonder that there is a complete absence of increase in brain or acoustic tumour risk; quite the contrary: most OR values (67-85% of 122 total ORs ≠ 1) were < 1, and the probability of this being chance is very low or almost zero (Figure 1D).

Since 2004, 17 case-control epidemiological studies have been published under the Interphone project launched by the International Agency for Research on Cancer (IARC) in 2000 [39-55], and overall are considered to lack any evidence for increase in head tumours in MP users. However, examination of all the above "negative" studies shows that there are bias, confounding factors, and errors in the methodological approach and the data processing and presentation. These factors include (see additional file 3):

• the low participation of cases or controls: ≤ 50% [43,44,46,50,54], ≤ 60% [45,52,55], ≤ 70% [39,42,47-49,53], not even given [37];

• the low percentages of exposed cases or controls: ≤ 30% [36-38], ≤ 40% [47,52], ≤ 50% [39,42,49,54], ≤ 60% [40,41,43,45,46,48,50,51,53,55], not even given [44];

• the low percentages of cases or controls exposed for ≥ 10 years: 0% [36-38,45,49,52,53], ≤ 5% [39,47,51,55], ≤ 10% [40-42,46,48], not even given [44];

• the inadequate definition for "normal use of cell phones" as "at least 1 phone call per week, for at least 6 months". Therefore, if a risk exists, it is "diluted" because of the dominance, in the examined sample, of subjects exposed too little: the average daily use of MPs in subjects considered "exposed" by Interphone is just 2-5 minutes per day, very scarcely representative of the intensive use made of cellphones today;

• the failure to include cordless users who, although exposed, are included among the non-exposed. The Interphone authors justify the exclusion of cordless users through the postulation that the intensity of the EM emission of this type of MP should be irrelevant and in any case much lower than emissions from cell phones, but in fact quite the reverse is true [1-3,9,10,56], to the extent that significant increases in the incidence of malign and benign brain tumours are found by Hardell also in those using only cordless phones (Table 1).

• the relative prevalence in controls exposed over the non-exposed subjects which is due to the fact that, there being no blind protocol, the subjects interviewed knew what was the purpose of the study. Therefore, MP users willingly elect to participate in the study, aware of its goals, while non-users tend to decline. This "selection bias" is recognized by the Interphone authors themselves, but in their view it does not cause reduction in OR of more than 10% [44,57], which is true for the overall Interphone data, but in some studies this bias alone can result in a more significant reduction in OR assessment: ≥ 15% [45,55], ≥ 25% [39,41,42], ≥ 30% [37,38,52,54], ≥ 50% [36] (see additional file 4).

In the negative cohort studies [58-61], where exposure is based simply on the fact that subjects work for an industry that produces MPs or are mobile telephony company subscribers (i.e. without the need to question participants), and where the illness/mortality incidence data estimate is based on linkage of data from people exposed with data from national tumour registers, there is an overabundance of data showing a reduced risk of those exposed - in all subjects [58,61] or only in males [59,60] - often s.s. and with very little probability of being chance (Figure 1E, see additional file 5). In these studies the s.s. reduction in risk even concerns organs that most certainly cannot be irradiated during the calls, in particular lung, stomach, liver and pancreas, and also the mortality from all causes, cardiac problems, liver cirrhosis, and car accidents. Clearly, the above "healthy worker effect" is due to systematic methodological errors and bias, e.g. the low prevalence of long exposures or latencies or the inadequate definition of cellphone use.

In the negative case-control studies [36-55] the combination of all the above factors leads to strong underestimation of the risk, and together act such that the majority of OR values are < 1, often s.s. (Figure 1B):

• in the 17 Interphone studies, out of 1084 OR values different from 1, 76% are < 1 and only 24% are > 1:

• the prevalence of OR values < 1 is extremely unusual: = 100% [44], ≥ 90% [41,54], ≥ 80% [45-47,49,52], ≥ 70% [39,42], ≥ 60% [43,48,51,55];

• the probability of this asymmetric distribution of OR being chance in 6 of these studies is low [39,42-44,48,51], while in another 5 [41,46,47,49,54], as in the overall data, it is practically zero;

• Lloyd-Morgan [62] applied a probability test to a distribution identical to that above, obtained by examining a lower number of OR values from 11 of the Interphone studies 76% OR < 1 and 24% OR > 1), and found the probability of this being chance to be 6.2 × 10^-20^;

• even more extraordinary, the OR values in 4 studies fall off with increased duration of exposure to MPs and/or latency time [36,39,48,52].

Discarding the idea of this being due to a protective effect from head tumour risk effected by MP use (not supported by experimental data - indeed, not even the Interphone authors support it), the only explanation can be found from a strong reduction in the assessment of risk resulting from the methodological errors present in the Interphone protocol.

The Interphone researchers themselves have published various studies on the methodological bias and flaws present in their work [44,57,63]. Most of the errors are attributed to the fact that the exposure is assessed on the basis of the data self-reported by participants in the case-control study ("recall errors"): in particular, it has been claimed that the increased risks reported in some studies (Table 2) could be due to cases blaming MPs as the cause of the disease. However, recently Hardell published the results of a case-control study on mortality (not incidence) due to malignant brain tumours in subjects who had used MPs and died before the interview could be performed, and found that use of analogue or digital cell-phones gave a s.s. increased risk, highest in the > 10 year latency group (OR = 2.4; 95%CI = 1.4-4.1), increasing with cumulative number of lifetime hours of cellular use and being highest in the > 2000 h group (OR = 3.4; 95% CI = 1.6-7.1) [64].

**Table 2:** Increased OR values in the Interphone studies on relationships between MP use and head tumours.

Author (tumour type)	year	**ref**.	years MP use	total tumours cases/controls and OR (95%CI)		ipsilateral tumours cases/controls and OR (95%CI)		contralateral tumours cases/controls and OR (95%CI)	
Lonn et al.	2004	40	since ≥ 10	14/29	1.9 (0.9-4.1)	12/15	3.9 (1.6-9.5)	4/17	0.8 (0.2-2.9)
(acoustic neuromas)			for ≥ 10	11/26	1.6 (0.7-3.6)	9/12	3.1 (1.2-8.4)	4/16	0.8 (0.2-3.1)
Schoemaker et al. 2005		43	since ≥ 10	47/212	1.0 (0.7-1.5)	31/124	1.3 (0.8-2.0)	20/105	1.0 (0.6-1.7)
(acoustic neuromas)			for ≥ 10	31/131	1.1 (0.7-1.8)	23/72	1.8 (1.1-3.1)	12/73	0.9 (0.5-1.8)
Lonn et al.	2005	41	since ≥ 10	25/38	0.9 (0.5-1.5)	15/18	1.6 (0.8-3.4)	11/25	0.7 (0.3-1.5)
(gliomas)			for ≥ 10	22/33	0.9 (0.5-1.6)	14/15	1.8 (0.8-3.9)	9/23	0.6 (0.3-1.4)
(meningiomas)			since ≥ 10	12/36	0.9 (0.4-1.9)	5/18	1.3 (0.5-3.9)	3/22	0.5 (0.1-1.7)
			for ≥ 10	8/32	0.7 (0.3-1.6)	4/15	1.4 (0.4-4.4)	3/23	0.5 (0.1-1.8)
Hepworth et al.	2006	46	since ≥ 10	66/112	0.9 (0.6-1.3)				
(gliomas)			for ≥ 10	48/67	1.14 (0.74-1.73)				
			regular use			278/486	1.24 (1.02-1.52)	199/491	0.75 (0.61-0.93)
Schuz et al.	2006	47	females only ≥ 0.5	30/38	1.96 (1.1-3.5)				
(gliomas)									
Lonn et al.	2006	48	since ≥ 10	7/15	1.4 (0.5-3.9)	6/9	2.6 (0.9-7.9)	1/9	0.3 (0.0-2.3)
(parotid gland tumours)			for ≥ 10	5/13	1.1 (0.4-3.6)	4/8	2.0 (0.5-7.0)	1/8	0.3 (0.0-2.6)
Klaeboe et al.	2007	49	since ≥ 6	70/73	0.8 (0.5-1.2)	39/37	1.3 (0.8-2.1)	32/42	0.8 (0.5-1.4)
(gliomas)			for ≥ 6	55/61	0.7 (0.4-1.2)	30/30	1.2 (0.7-2.1)	27/34	0.9 (0.5-1.5)
Lahkola et al.	2007	50	since ≥ 10	143/220	0.95 (0.74-1.23)	77/117	1.39 (1.01-1.92)	67/121	0.98 (0.71-1.37)
(gliomas)			for ≥ 10	88/134	0.94 (0.69-1.78)	43/74	1.14 (0.76-1.72)	41/71	1.01 (0.67-1.53)
Lahkola et al.	2008	54	since ≥ 10	73/212	0.91 (0.67-1.25)	33/113	1.05 (0.67-1.65)	24/117	0.62 (0.38-1.03)
(meningiomas)			for ≥ 10	42/130	0.85 (0.57-1.26)	21/73	0.99 (0.57-1.73)	13/68	0.64 (0.33-1.23)
Interphone	2010	72	≥ 1640 calls	160/113	1.82 (1.15-2.89)	100/62	1.96 (1.22-3.16)	39/31	1.25 (0.64-2.42)
(gliomas)									
Sadetzki et al.	2008	53	> 5479 calls	86/157	1.13 (0.79-1.61)	121/159	1.58 (1.11-2.24)	46/135	0.78 (0.51-1.19)
(parotid gland tumours)			> 266.3 hours	80/155	1.03 (0.72-1.47)	115/158	1.49 (1.05-2.13)	48/129	0.84 (0.55-1.28)
			> 5479 calls <5-year latency	47/82	1.16 (0.74-1.82)	35/40	1.80 (1.05-3.10)	12/41	0.63 (0.31-1.30)
"			> 5479 calls >5-year latency	120/215	1.08 (0.77-1.50)	86/119	1.50 (1.03-2.20)	34/94	0.84 (0.52-1.34)
only regular users			> 5479 calls	86/157	1.48 (1.05-2.10)				
"			> 18997 calls	81/140	1.51 (1.05-2.17)				
"			> 1035 cumulative calls	83/134	1.50 (1.04-2.16)				
			≥ 18997 calls, urban areas	49/99	1.00 (0.65-1.55)				
			" rural areas	32/41	1.81 (1.04-3.14)				
			≥ 1035 hours, urban areas	51/96	1.02 (0.67-1.58)				
			" rural areas	32/38	1.96 (1.11-3.44)				

95% s.s. data

### Hardell *versus *Interphone

The low number of cases with ≥ 10 years latency in the above negative studies is confirmed by data given in the last Interphone Study Results update [65]:

• only 54% of overall cases with "regular since ever use" (≥ 1 call/week for ≥ 6 months);

• only 5% of overall cases actually exposed for ≥ 10 years;

• only 2% of overall ipsilateral actually exposed for ≥ 10 years;

• while OR < 1 predominate in data referring to "regular use" of cell-phones (85%, of which 22% s.s.), the OR distribution clearly shifts towards values > 1 for only ipsilateral tumours with ≥ 10-years of cellular use or latency (86%, of which 25% s.s.), with the percentage of s.s. OR > 1 decreasing to 12% for total tumours and falling to 0% for contralateral tumours (Figure 1C, see additional file 6).

Moreover, in some of the Interphone studies s.s. increases in risk for ipsilateral tumours are quite common in people having used MPs since or for ≥ 10 years, and - more generally - even when there is no significant evidence of risk, a clear increase in OR values is often seen considering the figure for ipsilateral rather than total tumours, while there is a net fall for just contralateral tumours (Table 2). Taking into consideration the systematic underestimation of OR values in the Interphone studies, this is a clear indicator of probable carcinogenic risk.

The meta-analysis of Ahlbom [4] includes some of the US studies [36,37] and some of Hardell's earlier data (1999, 2001, 2002, not quoted in the present review) on risks of glioma, clearly lacking cases with ≥ 10-year latency time. Moreover it reports from Hardell [8] only data selectively chosen for subjects with "ever/never use" (> 5 year latency) but not, among those with 10 years since first use, the much more significant increases of risks, although these are clearly indicated in Hardell's paper analogue, digital, and cordless phone use. This meta-analysis shows data overall lacking any indication of carcinogenic risk, but underlines the absolute incompatibility between the two data sources: 83% of Hardell's risk data (OR) are > 1, 43% of which s.s., while the Interphone data are largely < 1 (73%), 11% of which s.s. (Figure 1C, see additional file 6).

**Figure 2:**
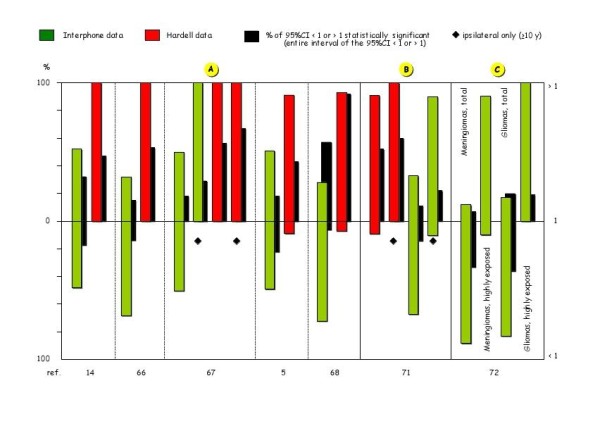
**Data from Hardell and Interphone meta-analyses: percentage of the OR values > 1 or < 1, and percentage of those statistically significant**.

In contrast, the meta-analyses of Hardell [14,66], Kundi [5], and Khurana [67] including the literature data on ipsilateral head tumours in people having used MPs since or for ≥10 years - and so also part of the Interphone data [40,43,46,50] - show large and s.s. increases (100%) for the risk of ipsilateral astrocytomas with high level of malignancy, and sizable and s.s. increases (50-140%) for the risk of acoustic neuromas (Table 3). These increases are smaller than those found by Hardell in the pooled analyses of his data alone (Table 1), being "diluted" with the Interphone data corresponding to the requirements indicated above. Indeed, by separating the overall OR data of these meta-analyses according to their source [5,14,66,68], only Hardell's OR data are systematically > 1 (90-100%), 50-90% of which are s.s., whereas Interphone data include 50-70% of OR < 1, a proportion of which (up to > 20%) are s.s. Moreover, when only ipsilateral data are considered [67], even 100% of the Interphone OR are > 1, 29% of which are s.s. (Figure 2A, see additional file 7).

**Table 3:** Results of the meta-analyses by Hardell, Kundi, and Kurana including Interphone data (≥ 10 year latency)

tumours		all		ipsilateral		contralateral	
astrocytomas I-IV	ref. 14, 66	(338/511):	1.2; 0.8-1.9	(n.s.):	2.0; 1.2-3.4	(n.s.):	1.1; 0.6-2.0
″	5	(233/330):	1.5; 1.2-1.8	(n.s.):	1.9; 1.4-2.4	-:	not analyzed
″	67	(233/330):	1.3; 1.1-1.6	(118/145):	1.9; 1.4-2.4	(93/150):	1.2; 0.9-1.7
							
neuromas	ref. 14, 66	(83/355):	1.3; 0.6-2.8	(53/167):	2.4; 1.1-5.3	(30/151):	1.2; 0.7-2.2
″	5	(67/311):	1.3; 0.95-1.9	(n.s.):	1.5; 1.1-2.5	-:	not analyzed
"	67	(67/311):	1.3; 0.97-1.9	(41/152):	1.6; 1.1-2.4	(26/134):	1.2; 0.4-1.03
							
meningiomas	ref. 14, 66	(61/152):	1.3; 0.9-1.8	(20/46):	1.7; 0.99-3.1	(15/52):	1.0; 0.3-3.1
″	5	(116/320):	1.1; 0.8-1.4	(n.s.):	1.3; 0.9-1.9	-:	not analyzed
	67	(116/320):	0.9; 0.7-1.3	(48/141):	1.1; 0.7-1.7	(36/164):	0.6; 0.4-1.03

95% s.s. data; n.s. = not specified

**Figure 3:**
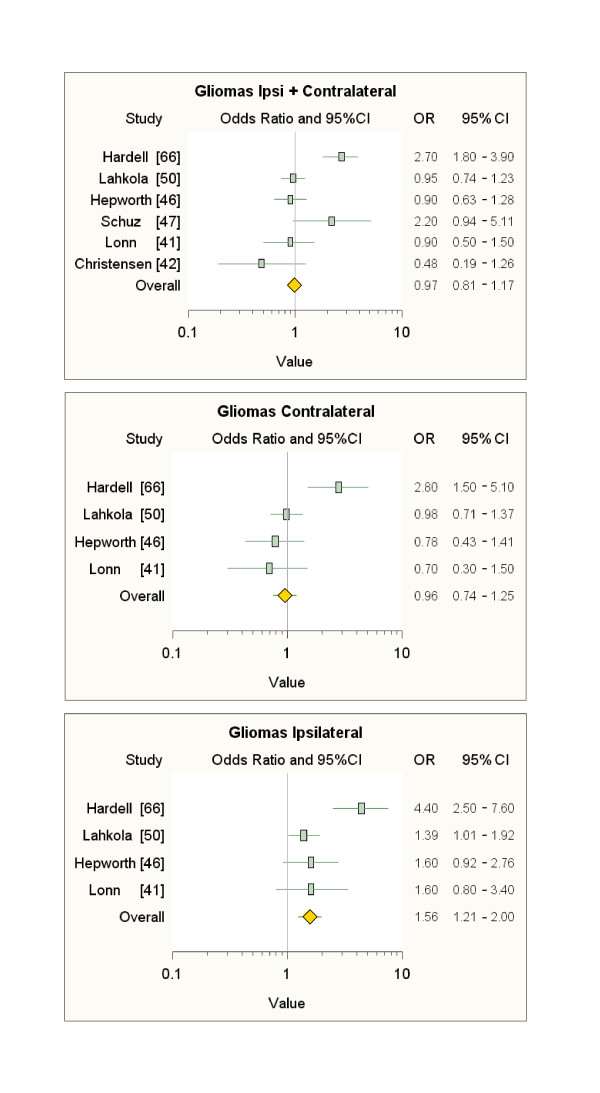
**Meta-analyses on data on gliomas after ≥ 10-year latency**.

**Figure 4:**
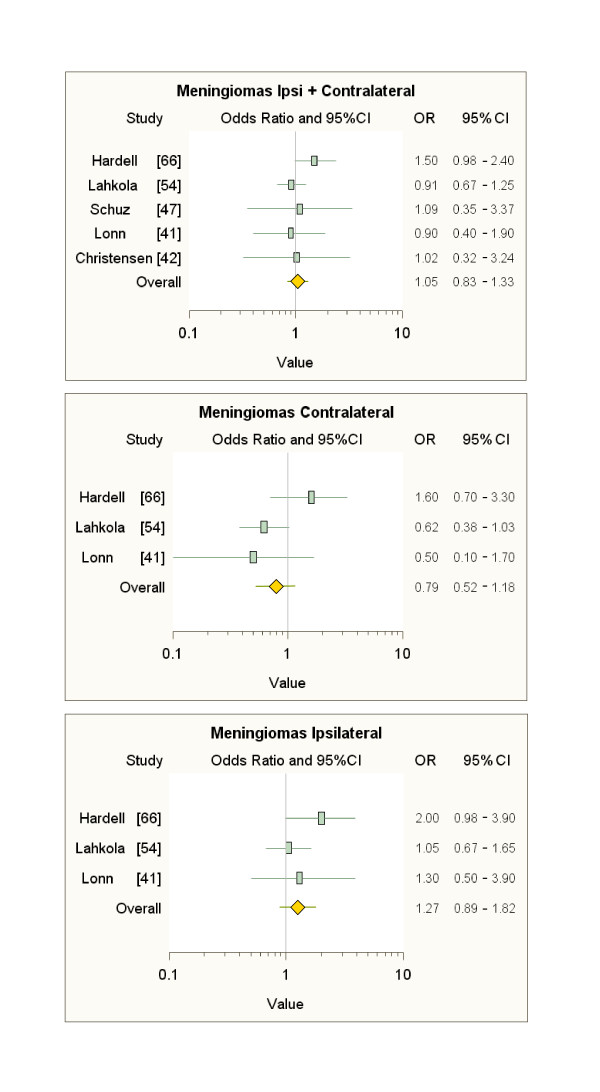
**Meta-analyses on data on meningiomas after ≥ 10-year latency**.

**Figure 5:**
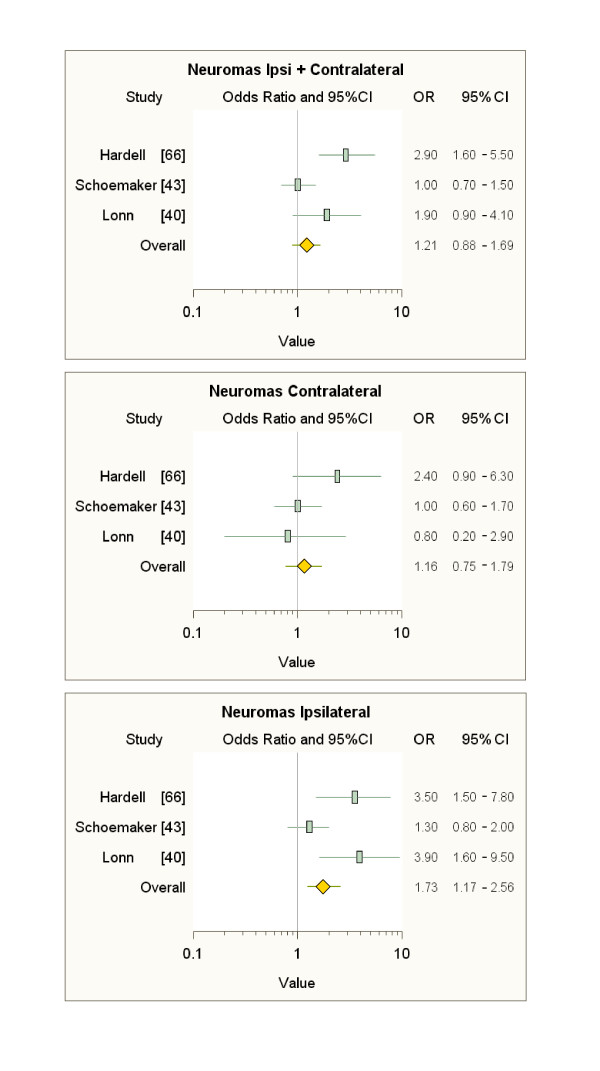
**Meta-analyses on data on acoustic neuromas after ≥ 10-year latency**.

Also our meta-analyses of the literature data (Figures 3, 4, 5), limited to subjects with ipsilateral tumours and MP latency ≥ 10 years (see additional file 8), show sizable and s.s. increases in risk of only ipsilateral acoustic neuromas (over 70%) and astrocytomas (almost 60%) compared to subjects not exposed to MP radiation, but it should be noted that the overall figure for these meta-analyses is strongly conditioned by the inclusion of the Interphone data. The results of our meta-analyses confirm the need to identify the head tumour localisation relative to the habitual head side of MP use, which is exposed to 97-99% of the radiation; therefore, the failure to identify the ipsilaterality of tumours adds an additional "dilution factor" to the risk evaluation.

Instead, the meta-analyses by Lloyd-Morgan [62] and Kan [69], limited to a subset of the Interphone data and without analysing tumour laterality or restricting reference to cases with MP use or latency ≥10 years, show a prevalence (75%) of OR values < 1, partly (33%) s.s. for the Interphone data, and an equal split of values < 1 and > 1 for Hardell's data, 100% of those > 1 being s.s (see additional file 7). The same pattern is shown by Lahkola meta-analysis [70], based on a "targeted" choice of data from the first US studies [36-38] and even from a cohort study [59], as well as from certain Interphone [41-43] and Hardell studies [8,9] (these latter data for the main part selectively chosen). In fact, Lahkola [70], besides including Hardell's earlier data (1999, 2002, not quoted in the present review) clearly lacking cases with ≥10-year latency time, calculated "through the pooling of different exposures or tumor categories" moderate risks for >5-year latency of neuromas plus meningiomas and of malign intracranial tumours from Hardell [8,9], whereas the original Hardell's much higher risks of meningiomas, neuromas, and malign brain tumours for >10 year latency were not included in Lahkola's meta-analysis.

The reasons underlying the discrepancy between Hardell's positive data and the negative Interphone findings are seen clearly by close examination of the latest articles from the two groups. Hardell [71] carried out a new meta-analysis, which took into account the Interphone data as well as his own: while the data overall do not show any increase in head tumour risk in MP users, limiting the meta-analysis to just ipsilateral tumours in individuals with ≥ 10-year latency, a s.s. increase in risk is found for gliomas (OR = 1.9; 95%CI = 1.4-2.4) and for acoustic neuromas (OR = 1.6; 95%CI = 1.1-2.4). Furthermore, while in Hardell's data > 90% of OR values are > 1, for the main part (>50%) s.s., this is the case for the Interphone data only when the analysis is limited to ipsilateral tumours in individuals with ≥ 10-year latency: 90% of OR values > 1, 22% of which s.s. (Figure 2B, see additional file 9).

In the first "official" Interphone Study Group [72], considering gliomas and meningiomas, the prevalence of OR values < 1 is notable (almost 80%), over 30% of these being s.s. (Figure 2C). Obviously, also this study is characterized by the usual bias and flaws:

• the low participation of cases (78% for meningiomas: range 56-92%; 64% for gliomas: range 36-92%), and especially of controls (53%: range 42-74%);

• the low median lifetime cumulative call time: 75 h for meningiomas (median: 2 h/month, i.e. 4 min/day), and 100 h for gliomas (median: 2.5 h/month, i.e. 5 min/day);

• the low percentage of cases with ≥ 10 y since the start of ipsilateral MP use: 3% of meningiomas, and 6.5% of gliomas.

However, analysis limited to subjects with "highest cumulative call times" shows a marked prevalence of OR values >1 [90% for meningiomas, and 100% for gliomas (20% s.s.)] (Figure 2C, see additional file 9). Moreover, given the selection bias due to the under-representation of never users among controls, an analysis was carried out with short-term users as controls. In this analysis, the OR values for glioma are almost all > 1, 30% of which s.s. with a dose-response relationship, showing that those who used MPs for ≥ 10 years are twice as likely to develop a brain tumour, especially in the ipsilateral side (OR = 1.96; 95%CI = 1.22-3.16) compared to total tumours (OR = 1.82; 95%CI = 1.15-2.89) and contralateral tumours (OR = 1.25; 95%CI = 0.64-2.42) (Table five of the Interphone text, and Table in its Appendix 2 online), just what is expected in the case of MPs having oncogenic action. This should rule out the possibility of the increase in risk of head tumours in high MP users, and indeed the increase in gliomas, acoustic neuromas and parotid gland tumours reported in some Interphone studies (Table 2), being due to methodological bias and confounding factors.

The conclusive report from Interphone [72] was accompanied by a commentary [73] whose title is very telling - "Call me on my mobile phone ... or better not? - a look at the Interphone study results", which pointed out some of the chief bias highlighted in the present report. Our analyses strongly reduces the uncertainty of the response to the quoted question: "better not" !

And indeed, even some Interphone authors have expressed disagreement with the reassuring interpretation of the Interphone results, which essentially indicates a lack of cause for alarm [72]. In September 2009, before the US Senate [74], Sadetzki defended the validity of her results showing an increase in risk of parotid tumour in strong cell-phone users, particularly in rural areas [53]. Finally, an editorial by Cardis, former Interphone coordinator, and by Sadetzki - also under a highly significant title "Indications of possible brain-tumour risk in mobilie-phone studies: should we be concerned?" [75] - gives a careful discussion of a selection of Hardell's main papers [1,2,10], noting that these show an increase in cerebral tumour risk in people using MPs for relatively long periods, and recognizes that the Interphone research contains a number of bias that lead to large underestimation of the risk values, among which some of those highlighted in the present report.

On the other hand, the editorial points out a number of observations supporting the risk:

• a 40% increase risk for glioma in the highest decile of cumulative call time;

• the increase of risk with time since start of use, suggesting a true effect of mobile-phone use;

• the increased risk of tumours in the temporal lobe in the highest decile of cumulative time.

The authors conclude that "the overall balance of the aboved-mentioned arguments suggests the existence of a possible association" between MP exposure and increased head tumour risk.

## Discussion

Previous studies identified a number of study design flaws and bias that give rise to underestimation of the real risk in epidemiological studies, particularly in those funded by industries [76,77]. The present paper, which concerns one of the most presently controversial debate - the possible relationship between MP use and increased risk of head tumours - shows that the negative results produced by studies funded by the cell-phone companies are affected by many biases and flaws, giving rise to a systematic underestimate of the risk. On the contrary, studies producing positive results - without errors and financial conditioning - indicate a cause/effect relationship supported by biological plausibility.

It must be noticed that the s.s. increase in malignant brain tumour risk repeatedly reported by Hardell among long-term MP users [1-3] is supported by the age-adjusted incidence increase of such tumours in Sweden [78]: during 1970-2007 the annual age-adjusted increase for all brain tumours was + 0.28% (95%CI = + 0.04 to + 0.52), whereas during 2000-2007 the figure for astrocytomas was + 1.55 (95% CI = - 0.15 to + 3.27, and even higher and s.s. in the age group > 19 years (+ 2.16; 95%CI = + 0.25 to 4.10). In addition, the s.s. increase in the risk of parotid gland malignant tumours reported by Sadetzky in cell-phone users [53] is supported by the incidence increase of such tumours in Israel [79]: the mean annual incidence of parotid cancers increased 4-fold from 1970 (16 cases/year) to 2006 (64 cases/year), whereas the incidence of other salivary gland tumours remained stable. The steepest increase in parotid cancers occurred after 2001, with an average of 37 cases annually before that date, and 61 cases per year subsequently; an increase of this magnitude cannot be due to population growth as the population of Israel increased 2.1-fold from 1970 to 2001, but only 1.1-fold from 2001 to 2006. The above data seem to indicate that, starting from 2000-2001, a new factor capable of increasing the risk of malignant head tumours among MP users began to manifest its effect, which is in accordance with the ≥ 10-15 years latency reached by cellular and cordless phone users in those years in both Sweden and Israel (see Background).

There are many bias and flaws in the Interphone and similar studies that lie behind the large prevalence of OR values < 1 in the overall results, giving rise to a systematic underestimate of the risk [78,80-84] whereas the protocol by Hardell producing positive results is without apparent errors or financial conditioning (Table 4), the results indicating a cause-effect relationship supported by biological plausibility [17-34]. A review on health effect of MPs showed that the studies reporting one or more s.s. positive results were funded by public bodies, while studies funded exclusively by industries were seven fold less likely to report at least one such result, and the difference between the two sets of data was highly s.s. [35]. According to the authors "this study indicates that the interpretation of the results from existing and future studies ... should take sponsorship into account".

**Table 4:** Errors in negative Interphone studies [4,36-55,65,72], and reliability of positive Hardell studies [1-3,7-10,64,71,78].

study, design, methods	negative studies	positive studies
Mobile phone use	inadequate: 2-5 min/day	significant: 16-32 min/day
Latency time	<5% cases with latency ≥10 y	>18% cases with latency ≥10 y
Cordless phone users	considered unexposed	considered exposed
Ipsilateral tumour latency	≥10 y for only 2% cases	≥10 y for >16% cases
Head tumours identified	only gliomas, meningiomas, neuromas, parotid tumours	also other head tumours types
Deceased cases	not included	included: proxy interviews
Interviews	not blind	always double blind
Type of interviews	face-to-face	mailed questionnaires
Time of interviews	cases: during hospitalisation	cases: after hospitalisation
	controls: at home	controls: at home
Exposure assessment	non blind interview	blind questionnaire
Data processing	not stated (not blind?)	Blind
Laterality attribution bias	present	Absent
Delayed interviews	for controls compared to cases	not delayed
Participation	reduced up to 40%	always near to 90%
Selection	exposed controls prevail	no selection bias
Documentation	positive data ignored	no documentation bias
Funding	co-funded by MP Companies	funded only by Public Bodies

Likewise, the discrepancy between the positive data of Hardell and the negative data from Interphone is highlighted by the authors that performed a random-effect model meta-analysis of 24 case-control studies [85]. These authors observed a s.s. positive association between MP use and increased head cancer risk in 10 studies ("high-quality studies", including 7 studies by Hardell, only 1 by Interphone, and 2 by other groups), whereas a negative association (i.e. an apparent "protective effect") was observed in 14 studies ("low-quality studies", including 12 by Interphone, and 2 by other groups). Elements in the method used to evaluate the "quality" of the studies were: a) blind or non-blind protocol; b) presence or absence of participation and selection bias of cases and controls; c) relevant or marginal MP exposure; d) adequate or inadequate latency or overall time of MP use; e) scrutiny of tumour laterality; f) funding by independent sources or by cell-phone companies. The authors make the following conclusion: "We feel the need to mention the funding sources for each research group because it is possible that these may have influenced the respective study designs and results".

The Hardell group was supported only by grants from Public Bodies, whereas the Interphone-related studies by the Quality of Life and Management of Living Resources program of the European Union and the International Union Against Cancer; but the latest received funds for those studies from the Mobile Manufacturers Forum and the Global System for Mobile Communication Association [86]. According to the Interphone protocol [86], "the partial funds provided by the above cell-phone Associations to the International Union Against Cancer complement funds from non-commercial sources including the European Union and national local research funding organization", but "provision of funds to the Interphone study investigators via the International Union Against Cancer is governed by agreements that guarantee Interphone's complete scientific independence", and "the funders of the Interphone studies do not have access to any results of the studies before their publication. They may, however, be informed, together with representatives of other concerned organizations such as consumer groups, a maximum of seven days before the publication of the results, under strict terms of confidentiality".

In addition to the above funds, several authors participating in the Interphone study received additional funding from their national MP companies [43,44,47,51,63] or by other private companies [42,59,60], such that a substantial portion of the Interphone Study funding came from the cell-phone industry. These additional funds are not specified in the Interphone protocol [86], and the agreements regulating access to the experimental results and the control of their use by the array of national cell-phone and other private companies involved are not known. Furthermore, other negative studies quoted in the present article have been supported by the mobile phone industry, for example the two Muscat studies [36,37] (Cellular Industry Telecommunications Association via the Wireless Technology Research) [19,62], the Johansen study [59] (TeleDanmark Mobil, Sonofon and the International Epidemiology Institute, a private company operating as a cell-phone industry adviser), and the Morgan study [58] (Motorola).

Nevertheless, of the authors of the above negative studies, 14 [36,37,39-44,46,50,53,54,58,59] do not make any declaration about conflict of interest, 3 [47,48,52] state "conflict of interest: none declared" (it is not clear whether this is from the authors or from the editor), while 4 [45,49,51,55] declare "conflict of interest: none".

Also the European Environment Agency [87], the European Parliament [88], and two recent papers [89,90] have expressed preoccupations about the effects on human health, particularly on that of young people, by the continuous RF exposure produced in public places and at home by wi-fi for internet access and MP use. The European Parliament has also pointed at "the need to evaluate scientific integrity of the authors, in order to forestall possible risks, conflicts of interest or even frauds which tend to arise in a context of growing competition among researchers" [88].

## Conclusions

Our examination of the literature data, together with the results of our and other's meta-analyses, lead to the conclusion that even today the risk of head tumours resulting from MP use is very high. Lloyd-Morgan, while underestimating by 50% the number of cell users, without considering cordless users and assuming a minimum latency time of 30 years, calculates "there would be about 1900 cell-phone-induced brain tumours out of about 50,000 brain tumours diagnosed in 2004, increasing to about 380,000 cell-phone-induced brain tumours within 2019 in the USA alone", which would require "an increase in health costs of an annual US$ 9.5 billion and the need for a 7-fold increase in number of neurosurgeons". An estimate of the incidence of head tumours must begin with the correct number of cell-phone users (5 billion subscriptions worldwide at mid 2010), should also consider the risk to cordless users, and assume at least a doubling of the incidence of head tumours and of acoustic neuromas as documented by Hardell already after a latency of at least 10-15 years.

Most likely, a number of factors raise our concern still further - for example, the latency of head tumour induced by MPs can exceed 30 years; risk is higher in those starting MP use when young and who have not yet accumulated 10 years of latency; there is a continued rise in MP use by youngsters, attracted to new offers from the MP companies (photography, listening to music, videophony, internet, etc.); the data of Hardell on the increase in other types of malign and benign head tumour- besides brain gliomas, astrocytomas, and acoustic neuromas - are for the main part today only indicative. Therefore, today we are evaluating just the tip of an iceberg, and will have to wait one or two decades before its real dimensions come to light. But it is clear that the analysis we have presented already shows a clear increase in tumour risk, and - if it proves even partly founded - the use of MPs could lead to a health crisis of dramatic proportion.

Furthermore, the recent editorial by Cardis and Sadetzky about the conclusive Interphone report states that "There are now more than 4 billion people, including children, using mobile phones. Even a small risk at the individual level could eventually result in a considerable number of tumours and become an important public-health issue. Simple and low-coast measures, such as the use of text messages, hands-free kits and/or the loud-speaker mode of the phone could substantially reduce exposure to the brain from mobile phones. Therefore, until definitive scientific answers are available, the adoption of such precautions, particularly among young people, is advisable".

While recognizing that mobile telephony is an outstanding technology of inestimable value, responsible science must raise awareness of the risks involved.

We thus conclude that already today there is sufficient epidemiological evidence to warrant application of the precautionary principle aimed at:

• setting exposure limits that are precautionary;

• limiting the spread of wireless technology in schools and highly frequented places (libraries, offices, hospital wards);

• providing accurate information about the risks from exposure to MPs, with low-cost voluntary options ("prudent avoidance") based on the caution in the use of MPs. A 10-point list of simple personal actions designed to substantially reduce the exposure to cell-phone radiation was produced by Viennese Medical Officers in 2006, adopted in the same year by the French Agency on Radiofrequencies http://www.sante-radiofrequences.org, by several study groups [[17-19], also http://www.devradavis.com.

• awareness-raising in schools through a campaign on the use of the various wireless transmission technologies;

• discouraging the use of MPs by minors under 14 years;

• epidemiological monitoring of the possible oncogenic action of home and workplace EM exposures.

## List of Abbreviations

EM: electromagnetic; IARC: International Agency for Research on Cancer; MP: mobile phone; OR: odds ratio; CI: confidence interval; s.s.: statistically significant.

## Competing interests

The authors declare that they have no competing interests.

## Authors' contributions

AGL and SG are responsible for the collection and analysis of data, and writing the manuscript. NM performed the meta-analyses. All the Authors contributed to the discussion of data and draw conclusions, and approved the final manuscript.

## Supplementary Material

Additional file 1 **MP use and tumours**. Main features of the case-control studies by Hardell et al. on the relationships between MP use and brain and acoustic nerve tumours and other types of tumours. Click here for file

Additional file 2 **Features of case-control studies before Interphone**. Main features of case-control studies performed before the Interphone project on the relationships betweeen MP use and brain and acoustic nerve tumours. Click here for file

Additional file 3 **Feature of case control studies by Interphone**. Main features of the case-control Interphone studies on the relationships between MP use and head tumours. Click here for file

Additional file 4 **Reduction of OR by selection bias**. Percent reduction of the OR estimation due to selection bias of cases and controls. Click here for file

Additional file 5 **MP and CNS tumours in cohort studies**. Main features of the cohort studies on the relationships between MP use and tumours or central nervous system (CNS) diseases. Click here for file

Additional file 6 **Data of Interphone update and Ahlbom review**. Main features of the Interphone update and the Ahlbom review on the data from case-control studies on the relationships between MP use and head tumours. Click here for file

Additional file 7 **Meta-analyses on MP and head tumours**. Main features of the meta-analyses of case-control studies on the relationships between MP use and head tumours. Click here for file

Additional file 8 **Studies included in the meta-analysis**. Summary of studies included in the meta-analysis (latency time ≥ 10 years. Click here for file

Additional file 9 **Risk on MP and head tumours in Hardell and Interphone studies**. Risk (OR) distribution in the latest Hardell and Interphone studies on the relationships between MP use and head tumours. Click here for file
