# Supplementary material for: Mobile phones and head tumours. The discrepancies in cause-effect relationships in the epidemiological studies - how do they arise?
Source: Environ Health. 2011 Jun 17;10:59. doi: 10.1186/1476-069X-10-59 (PMC3146917; doi:10.1186/1476-069X-10-59)
Supplement: Additional file 1 — MP use and tumours. Main features of the case-control studies by Hardell et al. on the relationships between MP use and brain and acoustic nerve tumours and other types of tumours. [file 1476-069X-10-59-S1.DOC]

**File 1** Main features of the case-control studies by Hardell et al. on the relationships between MP use and brain and acoustic nerve tumours (A) and other types of tumours (B).

Author year ref % partecipants n. and % exposed n. and % exposed ≥10 a. n. and % OR ≠1 n. and % 95%CI <1 n. and % 95%CI >1

(tumour type) cases controls cases controls cases controls < 1 > 1 stat. signif.➀ stat. signif.➀

on tot. OR <1 on tot. OR >1

**A *positive data***

––––––––––––––––––––––––––––––––––––––––––––––––––––––––––––––––––––––––––––––––––––––––––––––––––––––––––––––––––––––––––––––––––––––––––––––––––––––––––––––––––––––––––––

Hardell et al. 2003 7 88 91 1072 1047 71 54 29 138 0 39

(malign+benign head tumours) 75% 71% 7% 5% 17% 83% 0% 28%

Hardell et al. 2005 8 89 84 290 459 64 93 20 216 0 85

(benign head tumours) 70% 66% 22% 20% 8% 92% 0% 39%

Hardell et al. 2006 9 88 84 254 587 97 93 13 260 0 136

(malign head tumours) 80% 72% 38% 16% 5% 95% 0% 52%

Hardell et al. 2006 10 89 89 1106 1036 138 102 9 165 0 84

(malign+benign head tumours) 51% 48% 12% 10% 5% 95% 0% 51%

Hardell et al. 2006 2 88 89 672 1172 98 147 20 282 0 101

(benign head tumours) 54% 54% 8% 7% 7% 93% 0% 36%

Hardell et al. 2006 1 90 89 583 1172 134 147 15 208 0 81

(malign head tumours) 64% 54% 15% 7% 7% 93% 0% 39%

Hardell and Carlberg 2009 3 88 89 1255 1172 232 147 11 117 0 39

(malign+benign head tumours) 58% 54% 11% 7% 9% 91% 0% 43%

––––––––––––––––––––––––––––––––––––––––––––––––––––––––––––––––––––––––––––––––––––––––––––––––––––––––––––––––––––––––––––––––––––––––––––––––––––––––––––––––––––––––––––

total: only pooled analyses (1, 2) 1255 1172 232 147 35 490 0 182

58% 54% 18% 13% 7% 93% 0% 37%

**B *additional data***

––––––––––––––––––––––––––––––––––––––––––––––––––––––––––––––––––––––––––––––––––––––––––––––––––––––––––––––––––––––––––––––––––––––––––––––––––––––––––––––––––––––––––––

Hardell et al. 2004 11 91 90 215 859 30 193 40 39 0 0

(salivary gland tumours) 81% 82% 14% 22% 51% 49% 0% 0%

Hardell et al. 2005 13 91 92 607 695 318 398 49 134 0 5

(non-Hodgkin lymphomas) 67% 68% 52% 57% 27% 73% 0% 4%

Hardell et al. 2007 12 91 89 373 475 28 18 79 117 5 8

(testicular cancers) 42% 55% 8% 4% 40% 60% 6% 7%

––––––––––––––––––––––––––––––––––––––––––––––––––––––––––––––––––––––––––––––––––––––––––––––––––––––––––––––––––––––––––––––––––––––––––––––––––––––––––––––––––––––––––––

- 95%CI superior limit < 1 for OR<1, and 95%CI inferior limit > 1 for OR>1
