# Supplementary material for: Mobile phones and head tumours. The discrepancies in cause-effect relationships in the epidemiological studies - how do they arise?
Source: Environ Health. 2011 Jun 17;10:59. doi: 10.1186/1476-069X-10-59 (PMC3146917; doi:10.1186/1476-069X-10-59)
Supplement: Additional file 2 — Features of case-control studies before Interphone. Main features of case-control studies performed before the Interphone project on the relationships betweeen MP use and brain and acoustic nerve tumours. [file 1476-069X-10-59-S2.DOC]

**File 2** Main features of case-control studies performed before the Interphone project on the relationships betweeen MP use and brain and acoustic nerve tumours.

Author year ref. % partecipants n. and % exposed n. and % esposed ≥10 y n. and % OR ≠1 n. and % 95%CI <1 n. and % 95%CI >1

(tumour type) cases controls cases controls cases controls < 1 > 1 stat. signif.➀ stat. signif.➀

*funding source* on tot. OR <1 on tot. OR >1

––––––––––––––––––––––––––––––––––––––––––––––––––––––––––––––––––––––––––––––––––––––––––––––––––––––––––––––––––––––––––––––––––––––––––––––––––––––––––––––––––––––––

Muscat et al. 2000 36 82 90 66 76 0 0 17 3 0 0

(head cancers) 14% 28% 0% 0% 85% 15% 0% 0%

*Cellphone Companies*

Inskip et al. 2001 38 93 86 161 202 0 0 77 19 0 0

(head cancers) 20% 27% 0% 0% 80% 20% 0% 0%

*no indication*

Muscat et al. 2002 37 n.r. n.r. 18 23 0 0 4 2 0 0

(acoustic neuromas) 20% 27% 0% 0% 67% 33% 0% 0%

*Cellphone Companies*

––––––––––––––––––––––––––––––––––––––––––––––––––––––––––––––––––––––––––––––––––––––––––––––––––––––––––––––––––––––––––––––––––––––––––––––––––––––––––––––––––––––––

- 95%CI superior limit < 1 for OR<1, and 95%CI inferior limit > 1 for OR>1; n.r. = not reported.
