# Supplementary material for: Mobile phones and head tumours. The discrepancies in cause-effect relationships in the epidemiological studies - how do they arise?
Source: Environ Health. 2011 Jun 17;10:59. doi: 10.1186/1476-069X-10-59 (PMC3146917; doi:10.1186/1476-069X-10-59)
Supplement: Additional file 3 — Feature of case control studies by Interphone. Main features of the case-control Interphone studies on the relationships between MP use and head tumours. [file 1476-069X-10-59-S3.DOC]

**File 3** Main features of the case-control Interphone studies on the relationships between MP use and head tumours.

Author year ref. % partecipants n. and % exposed n. and % exposed ≥10 y n. and % OR ≠1 n. & % 95%CI <1 n. & % 95%CI >1

(tumour type) cases controls cases controls cases controls < 1 > 1 stat. signif.➀ stat. signif.➀

on tot. OR <1 on tot. OR >1

–––––––––––––––––––––––––––––––––––––––––––––––––––––––––––––––––––––––––––––––––––––––––––––––––––––––––––––––––––––––––––––––––––––––––––––––––––––––––––––––––––––––––––

Christensen et al. 2004 ② 39 82 64 61 147 2 15 13 4 1 0

(acoustic neuromas) 49% 56% 3% 10% 77% 23% 0,8% 0%

Lonn et al. 2004 40 93 72 89 356 14 29 16 16 0 2

(acoustic neuromas) 60% 59% 16% 8% 50% 50% 0% 12,5%

Lonn et al. 2005 41 78 71 327 780 30 65 180 15 15 0

(gliomas, meningiomas) 51% 59% 9% 8% 92% 8% 8,3% 0%

Christensen et al. 2005 42 72 64 244 560 20 39 40 11 4 0

(gliomas, meningiomas) 48% 56% 8% 7% 78% 22% 10% 0%

Schoemaker et al. 2005 43 82 48 360 1934 47 212 35 17 1 1

(acoustic neuromas) 53% 55% 13% 11% 67% 33% 2,8% 5,9%

Lahkola et al. 2005 44 84 42 ③ 7 0 5 0

(head tumours) n.r. n.r n.r. n.r. 100% 0% 71% 0%

Takebayashi et al. 2006 45 84 52 51 192 0 0 9 2 0 0

(acoustic neuromas) 53% 58% 0% 0% 82% 18% 0% 0%

Hepworth et al. 2006 46 51 45 505 896 48 67 31 6 2 1

(gliomas) 52% 52% 10% 7% 84% 16% 6,4% 16,7%

Schuz et al. 2006 47 84 61 229 492 17 20 44 10 1 0

(gliomas, meningiomas) 31% 33% 7% 4% 81% 19% 2,3% 0%

Lonn et al. 2006 ② 48 82 70 101 587 6 51 32 15 0 0

(parotid gland tumours) 59% 60% 6% 9% 68% 32% 0% 0%

Klaeboe et al. 2007 49 74 69 279 675 0 0 92 11 9 0

(acoustic neuromas, gliomas, meningiomas) 52% 50% 0% 0% 89% 11% 14,7% 0%

Lahkola et al. 2007 50 60 50 869 1854 143 220 67 16 37 1

(gliomas) 58% 59% 16% 12% 81% 19% 55% 6,3%

Hours et al. 2007 51 73 75 188 257 3 1 69 44 2 0

(acoustic neuromas, gliomas, meningiomas) 54% 56% 2% 0,4% 61% 39% 2,9% 0%

Schlehofer et al. 2007 ② 52 89 53 28 73 0 3 8 2 1 0

(acoustic neuromas) 29% 38% 0% 4% 80% 20% 12,5% 0%

Sadetzki et al. 2007 53 87 66 570 1372 0 0 75 63 0 6

(salivary and parotid glands tumours) 62% 54% 0% 0% 54% 46% 0% 10%

Lahkola et al. 2008 54 74 50 573 1696 73 212 62 2 27 0

(meningiomas) 47% 58% 13% 13% 97% 3% 43,5% 0%

Takebayashi et al. 2008 55 71 51 173 329 11 10 49 33 0 0

(gliomas, meningiomas, pituitary adenomas) 55% 59% 8% 3% 60% 40% 0% 0%

–––––––––––––––––––––––––––––––––––––––––––––––––––––––––––––––––––––––––––––––––––––––––––––––––––––––––––––––––––––––––––––––––––––––––––––––––––––––––––––––––––––––––––

Total of studies: 4647 12200 414 944 829 255 105 11

52% 54% 9% 8% 76% 24% 13% 4%

- 95%CI superior limit < 1 for OR<1, and 95%CI inferior limit > 1 for OR>1

② ORs decrease together with the increase of exposition and/or latency.

③ Not reported.
