# Supplementary material for: Mobile phones and head tumours. The discrepancies in cause-effect relationships in the epidemiological studies - how do they arise?
Source: Environ Health. 2011 Jun 17;10:59. doi: 10.1186/1476-069X-10-59 (PMC3146917; doi:10.1186/1476-069X-10-59)
Supplement: Additional file 4 — Reduction of OR by selection bias. Percent reduction of the OR estimation due to selection bias of cases and controls. [file 1476-069X-10-59-S4.DOC]

**File 4 Percent reduction of the OR estimation due to selection bias of cases and controls.**

author year ref. OR calculation % reduc.

Muscat 2000 36 OR = 14 / 86 x 72 / 28 = 0.42 (-58%)

Inskip 2001 38 OR = 20 / 80 x 76 / 24 = 0.79 (-21%)

Muscat 2002 37 OR = 20 / 80 x 73 / 27 = 0.68 (-32%)

Interphone overall file 3 OR = 52 / 48 x 46 / 54 = 0.92 (-8%)

Christensen 2004 39 OR = 49 / 51 x 44 / 56 = 0.75 (-25%)

Lonn 2005 41 OR = 51 / 49 x 41 / 59 = 0.72 (-28%)

Christensen 2005 42 OR = 48 / 52 x 44 / 56 = 0.73 (-27%)

Takebajashi 2006 45 OR = 53 / 47 x 42 / 58 = 0.82 (-18%)

Schlehofer 2007 52 OR = 29 / 71 x 62 / 38 = 0.67 (-33%)

Lahkola 2008 54 OR = 47 / 53 x 42 / 58 = 0.64 (-36%)

Takebajashi 2008 55 OR = 55 / 45 x 41 / 59 = 0.85 (-15%)
