# Supplementary material for: Mobile phones and head tumours. The discrepancies in cause-effect relationships in the epidemiological studies - how do they arise?
Source: Environ Health. 2011 Jun 17;10:59. doi: 10.1186/1476-069X-10-59 (PMC3146917; doi:10.1186/1476-069X-10-59)
Supplement: Additional file 5 — MP and CNS tumours in cohort studies. Main features of the cohort studies on the relationships between MP use and tumours or central nervous system (CNS) diseases. [file 1476-069X-10-59-S5.DOC]

**File 5** Main features of the cohort studies on the relationships between MP use and tumours or central nervous system (CNS) diseases.

Author year n. and % cases n. and % cases n. and % SIR-SHR② n. & % 95%CI <1 n. & % 95%CI >1

(tumour type) ref. cases exposed exposed ≥10 y ➀ < 1 > 1 stat. signif.③ stat. signif.③

*funding source* on SIR-SHR <1 on SIR-SHR >1

––––––––––––––––––––––––––––––––––––––––––––––––––––––––––––––––––––––––––––––––––––––––––––––––––––––––––––––––––––––––––––––––––––––––––––––––––––––––––––––––––––––

Morgan et al. 2000 58 195,775 54,540 125 110 20 35 0

(head cancers and haemopoietic system) 28% 0.2% 85% 15% 32%

*Motorola*

Johansen et al. 2001 59 males 21 = 88% 3 = 12% 7 = 33% 0

(all tumours) 420,095 ➃ 420,095 56,648 = 13%

*Cellphone Companies* females 6 = 27% 16 = 73% 0 0

Schuz et al. 2006 60 males 15 = 79% 4 = 21% 9 = 47% 0

(all tumours) 420,095 ➃ 420095 56648 = 13%

*Public and Private Agencies* females 7 = 39% 11 = 61% 0 3 = 27%

Schuz et al. 2009 61 420,095 ➃ 420,095 221 26 7 16 0

(CNS diseases) 0,05% 79% 21% 62%

*Public and Private Agencies*

––––––––––––––––––––––––––––––––––––––––––––––––––––––––––––––––––––––––––––––––––––––––––––––––––––––––––––––––––––––––––––––––––––––––––––––––––––––––––––––––––––––

➀ From the beginning of contracts with telephone companies [59-61] or employment at Motorola [58].

② SIR, Standardized Incidence Ratio; SHR, Standardized Hospitalization Ratio.

③ 95%CI superior limit < 1 for OR<1, and 95%CI inferior limit > 1 for OR>1.

➃ Based on contracts with telephone companies.
