# Supplementary material for: Mobile phones and head tumours. The discrepancies in cause-effect relationships in the epidemiological studies - how do they arise?
Source: Environ Health. 2011 Jun 17;10:59. doi: 10.1186/1476-069X-10-59 (PMC3146917; doi:10.1186/1476-069X-10-59)
Supplement: Additional file 6 — Data of Interphone update and Ahlbom review. Main features of the Interphone update and the Ahlbom review on the data from case-control studies on the relationships between MP use and head tumours. [file 1476-069X-10-59-S6.DOC]

**File 6** Main features of the Interphone update [65] and the Ahlbom review [4] on the data from case-control studies on the relationships between MP use and head tumours

year use or reference data n. and % OR ≠1 n. and % 95%CI <1 n. and % 95%CI >1

<1 >1 stat. signif. ➀ stat. signif. ➀

on tot. OR <1 on tot. OR >1

***Interphone reviews***

(tumour type)

----------------------------------------------------------------------------------------------------------------------------------------------------

update 2008 regular since ever 23 4 5 0

(head tumours) 85% 15% 22% 0%

since ≥ 10 years 15 9 0 0

63% 37% 0% 0%

ipsilateral since ≥ 10 years 2 12 0 3

14% 86% 0% 25%

contralateral since ≥ 10 years 10 1 0 0

91% 9% 0% 0%

total 50 26 5 3

66% 34% 10% 12%

Ahlbom et al. 2009 Interphone 106 40 11 0

73% 27% 11% 0%

(head tumours)

Hardell 14 70 0 30

17% 83% 0% 43%

----------------------------------------------------------------------------------------------------------------------------------------------------

- 95%CI superior limit < 1 for OR < 1, and 95%CI inferior limit > 1 for OR > 1.
