# Supplementary material for: Mobile phones and head tumours. The discrepancies in cause-effect relationships in the epidemiological studies - how do they arise?
Source: Environ Health. 2011 Jun 17;10:59. doi: 10.1186/1476-069X-10-59 (PMC3146917; doi:10.1186/1476-069X-10-59)
Supplement: Additional file 7 — Meta-analyses on MP and head tumours. Main features of the meta-analyses of case-control studies on the relationships between MP use and head tumours. [file 1476-069X-10-59-S7.DOC]

**File 7** Main features of the meta-analyses of case-control studies on the relationships between MP use and head tumours.

n. and % OR ≠1 n. & % 95%CI < 1 n. & % 95%CI > 1

Author year ref. data examined < 1 > 1 stat. signif. ➀ stat. signif. ➀

on tot. OR < 1 on tot. OR > 1

––––––––––––––––––––––––––––––––––––––––––––––––––––––––––––––––––––––––––––––––––––––––––––––––––––––––––––––––––––––––––––––––––––––––––––––––

**Hardell** et al. 2007 14 Interphone ➁ 23 25 4 8

48% 52% 17% 32%

" " Hardell 0 19 0 9

0% 100% 0% 47%

**Hardell** et al. 2008 66 Interphone ➁ 28 13 4 2

68% 32% 14% 15%

" " Hardell 0 17 0 9

0% 100% 0% 53%

**Khurana** et al. 2009 67 Interphone ➂ 11 11 0 2

50% 50% 0% 18%

" " Interphone only ipsilateral 0 7 0 2

0% 100% 0% 29%

" " Hardell 0 9 0 5

0% 100% 0% 56%

" " Hardell only ipsilateral 0 3 0 2

0% 100% 0% 67%

**Kundi** 2009 5 Interphone ➁ 27 28 6 5

49% 51% 22% 18%

" " Hardell 5 49 0 21

9% 91% 0% 43%

**Kan** et al. 2008 69 Interphone ➁ 11 6 5 2

65% 35% 45% 33%

**Han** et al. 2009 68 Interphone ➁ 18 7 1 4

72% 28% 6% 57%

" " Hardell 1 13 0 12

7% 93% 0% 92%

**Myung** et al. 2009 71 Interphone ➁ 21 3 7 1

88% 12% 88% 12%

" Hardell 1 21 0 9

5% 95% 0% 100%

**Lloyd**-**Morgan** 2009 59 Interphone ➁ 15 7 3 0

68% 32% 20% 0%

**Lahkola** et al. 2006 70 Interphone ➁ 6 2 2 0

(Interphone) 75% 25% 33% 0%

" " Hardell 2 2 ­0 2

50% 50% 0% 100%

––––––––––––––––––––––––––––––––––––––––––––––––––––––––––––––––––––––––––––––––––––––––––––––––––––––––––––––––––––––––––––––––––––––––––––––––

➀ IC95% superior limit < 1 for OR < 1, or IC95% inferior limit > 1 for OR > 1; ➁ Some case-control studies preceeding Interfone ones are included; ➂ At least 10 year exposure or latency, and laterality correlation.
