# Supplementary material for: Mobile phones and head tumours. The discrepancies in cause-effect relationships in the epidemiological studies - how do they arise?
Source: Environ Health. 2011 Jun 17;10:59. doi: 10.1186/1476-069X-10-59 (PMC3146917; doi:10.1186/1476-069X-10-59)
Supplement: Additional file 8 — Studies included in the meta-analysis. Summary of studies included in the meta-analysis (latency time ≥ 10 years. [file 1476-069X-10-59-S8.DOC]

**File 8 Summary of studies included in the meta-analysis (latency time ≥10 years)**

| **Study** | **Study**  **period** | **Study countries** | **Age (years)** | **Tumor type** | | **Laterality** | **N. cases/ controls** | **OR**  **(95%CI)** |
| --- | --- | --- | --- | --- | --- | --- | --- | --- |
| Hardell, 2008 [66] | 1997-2003 | Sweden | 20-80 | Gliomas | | Ipsi-lateral | 41/28 | 4.4  (2.5-7.6) |
| Lahkola, 2007 [50] | 2000-2004 | Denmark, Finland, Norway, Sweden, U.K. | 20-69 Nordic countries  18-59 U.K. | Gliomas | | Ipsi-lateral | 77/117 | 1.39  (1.01-1.92) |
| Hepworth, 2006 [46] | 2000-2004 | U.K. | 18-69 | Gliomas | | Ipsi-lateral | n.a. | 1.60  (0.92-2.76) |
| Lonn,  2005 [41] | 2000-2002 | Sweden | 20-69 | Gliomas | | Ipsi-lateral | 15/18 | 1.6  (0.8-3.4) |
| **Meta-analysis (Test for heterogeneity χ^2^=1.27 p-value=0.005, I^2^=74%, H^2^=4.23** | | | | | | | **n.c.** | **1.56**  **(1.21-2.00)** |
| Hardell, 2008 [66] | 1997-2003 | Sweden | 20-80 | Gliomas | | Contralateral | 26/29 | 2.8  (1.5-5.1) |
| Lahkola, 2007 [50] | 2000-2004 | Denmark, Finland, Norway, Sweden, UK | 20-69 Nordic countries  18-59 U.K. | Gliomas | | Contralateral | 67/121 | 0.98  (0.71-1.37) |
| Hepworth, 2006 [46] | 2000-2004 | U.K. | 18-69 | Gliomas | | Contralateral | n.a. | 0.78  (0.43-1.41) |
| Lonn,  2005 [41] | 2000-2002 | Sweden | 20-69 | Gliomas | | Contralateral | 11/25 | 0.7  (0.3-1.5) |
| **Meta-analysis (Test for heterogeneity χ^2^=11.9 p-value=0.008, I^2^=73%, H^2^=3.97** | | | | | | | **n.c.** | **0.96**  **(0.74-1.25)** |
| Hardell, 2008 [66] | 1997-2003 | Sweden | 20-80 | Gliomas | Ipsi+contralateral | | 78/99 | 2.7  (1.8-3.9) |
| Lahkola, 2007 [50] | 2000-2004 | Denmark, Finland, Norway, Sweden, UK | 20-69 Nordic countries  18-59 U.K. | Gliomas | Ipsi+contralateral | | n.a. | 0.95  (0.74-1.23) |
| Hepworth, 2006 [46] | 2000-2004 | U.K. | 18-69 | Gliomas | Ipsi+contralateral | | 66/112 | 0.9  (0.63-1.28) |
| Schuz,  2006 [47] | 2000-2003 | Germany | 30-69 | Gliomas | Ipsi+contralateral | | 12/11 | 2.2  (0.94-5.11) |
| Lonn,  2005 [41] | 2000-2002 | Sweden | 20-69 | Gliomas | Ipsi+contralateral | | 25/38 | 0.9  (0.5-1.5) |
| Christensen, 2005 [42] | 2000-2002 | Denmark | 20-69 | Gliomas | Ipsi+contralateral | | 8/22 | 0.48  (0.19-1.26) |
| **Meta-analysis (Test for heterogeneity χ^2^=28.9 p-value<0.0001, I^2^=83%, H^2^=9.66** | | | | | | | **n.c.** | **0.97**  **(0.81-1.17)** |

| **Study** | **Study**  **period** | **Study countries** | **Age (years)** | **Tumor type** | **Laterality** | **N. cases/ controls** | **OR**  **(95%CI)** |
| --- | --- | --- | --- | --- | --- | --- | --- |
| Hardell, 2008 [66] | 1997-2003 | Sweden | 20-80 | Neuromas | Ipsi-lateral | 10/28 | 3.5  (1.5-7.8) |
| Schoemaker, 2005 [43] | 1999-2004 | Denmark, Finland, Norway, Sweden, UK | 20-69 Nordic countries  18-59 U.K. | Neuromas | Ipsi-lateral | 31/124 | 1.3  (0.8-2.0) |
| Lonn,  2004 [40] | 2000-2002 | Sweden | 20-69 | Neuromas | Ipsi-lateral | 12/15 | 3.9  (1.6-9.5) |
| **Meta-analysis (Test for heterogeneity χ^2^=7.24 p-value=0.027, I^2^=72%, H^2^=3.61** | | | | | | **53/167** | **1.73**  **(1.17-2.56)** |
| Hardell, 2008 [66] | 1997-2003 | Sweden | 20-80 | Neuromas | Contralateral | 6/29 | 2.4  (0.9-6.3) |
| Schoemaker, 2005 [43] | 1999-2004 | Denmark, Finland, Norway, Sweden, UK | 20-69 Nordic countries  18-59 U.K. | Neuromas | Contralateral | 20/105 | 1.0  (0.6-1.7) |
| Lonn,  2004 [40] | 2000-2002 | Sweden | 20-69 | Neuromas | Contralateral | 4/17 | 0.8  (0.2-2.9) |
| **Meta-analysis (Test for heterogeneity χ^2^=2.75 p-value=0.25, I^2^=27%, H^2^=1.37** | | | | | | **30/151** | **1.16**  **(0.75-1.79)** |
| Hardell, 2008 [66] | 1997-2003 | Sweden | 20-80 | Neuromas | Ipsi+contralateral | 20/99 | 2.9  (1.6-5.5) |
| Schoemaker, 2005 [43] | 1999-2004 | Denmark, Finland, Norway, Sweden, UK | 20-69 Nordic countries  18-59 U.K. | Neuromas | Ipsi+contralateral | 47/212 | 1.0  (0.7-1.5) |
| Lonn,  2004 [40] | 2000-2002 | Sweden | 20-69 | Neuromas | Ipsi+contralateral | 14/29 | 1.9  (0.9-4.1) |
| **Meta-analysis (Test for heterogeneity χ^2^=8.94 p-value=0.01, I^2^=78%, H^2^=4.47** | | | | | | **81/340** | **1.21**  **(0.88-1.69)** |

| **Study** | **Study**  **period** | **Study countries** | **Age (years)** | **Tumor type** | **Laterality** | **N. cases/ controls** | **OR**  **(95%CI)** |
| --- | --- | --- | --- | --- | --- | --- | --- |
| Hardell, 2008 [66] | 1997-2003 | Sweden | 20-80 | Meningiomas | Ipsi-lateral | 15/28 | 2.0  (0.98-3.9) |
| Lahkola, 2008 [54] | 2000-2004 | Denmark, Finland, Norway, Sweden, UK | 20-69 Nordic countries  18-59 U.K. | Meningiomas | Ipsi-lateral | 33/113 | 1.05  (0.67-1.65) |
| Lonn,  2005 [41] | 2000-2002 | Sweden | 20-69 | Meningiomas | Ipsi-lateral | 5/18 | 1.3  (0.5-3.9) |
| **Meta-analysis (Test for heterogeneity χ^2^=2.34 p-value=0.31, I^2^=15%, H^2^=1.17** | | | | | | **53/159** | **1.27**  **(0.89-1.82)** |
| Hardell, 2008 [66] | 1997-2003 | Sweden | 20-80 | Meningiomas | Contralateral | 12/29 | 1.6  (0.7-3.3) |
| Lahkola, 2008 [54] | 2000-2004 | Denmark, Finland, Norway, Sweden, UK | 20-69 Nordic countries  18-59 U.K. | Meningiomas | Contralateral | 24/117 | 0.62  (0.38-1.03) |
| Lonn,  2005 [41] | 2000-2002 | Sweden | 20-69 | Meningiomas | Contralateral | 3/23 | 0.5  (0.1-1.7) |
| **Meta-analysis (Test for heterogeneity χ^2^=4.47 p-value=0.11, I^2^=55%, H^2^=2.23** | | | | | | **39/169** | **0.79**  **(0.52-1.18)** |
| Hardell, 2008 [66] | 1997-2003 | Sweden | 20-80 | Meningiomas | Ipsi+contralateral | 38/99 | 1.5  (0.98-2.4) |
| Lahkola, 2008 [54] | 2000-2004 | Denmark, Finland, Norway, Sweden, UK | 20-69 Nordic countries  18-59 U.K. | Meningiomas | Ipsi+contralateral | 73/212 | 0.91  (0.67-1.25) |
| Schuz,  2006 [47] | 2000-2003 | Germany | 30-69 | Meningiomas | Ipsi+contralateral | 5/9 | 1.09  (0.35-3.37) |
| Lonn,  2005 [41] | 2000-2002 | Sweden | 20-69 | Meningiomas | Ipsi+contralateral | 12/36 | 0.9  (0.4-1.9) |
| Christensen, 2005 [42] | 2000-2002 | Denmark | 20-69 | Meningiomas | Ipsi+contralateral | 6/8 | 1.02  (0.32-3.24) |
| **Meta-analysis (Test for heterogeneity χ^2^=3.39 p-value=0.49, I^2^=0%, H^2^=0.89** | | | | | | **134/364** | **1.05**  **(0.83-1.33)** |

n.a.=not available; n.c.=not computed since sample size was not present in one publication.
