# Supplementary material for: Mobile phones and head tumours. The discrepancies in cause-effect relationships in the epidemiological studies - how do they arise?
Source: Environ Health. 2011 Jun 17;10:59. doi: 10.1186/1476-069X-10-59 (PMC3146917; doi:10.1186/1476-069X-10-59)
Supplement: Additional file 9 — Risk on MP and head tumours in Hardell and Interphone studies. Risk (OR) distribution in the latest Hardell and Interphone studies on the relationships between MP use and head tumours. [file 1476-069X-10-59-S9.DOC]

**File 9** Risk (OR) distribution in the latest Hardell et al. study (2009) [71] and The Interphone Study Group (2010) [72] on the relationships between MP use and head tumours.

n. and % OR ≠1 n. & % 95%CI <1 n. & 5 95%CI >1

study data tumour type cases < 1 > 1 stat. signif.➀ stat. signif.➀

on tot. OR <1 on tot. OR >1

-----------------------------------------------------------------------------------------------------------------------

Hardell Hardell head tumours ② total 2 21 0 11

9% 91% 0% 52%

ipsilateral ③ 0 5 0 3

0% 100% 0% 60%

Interphone head tumours ② total 37 18 5 2

67% 33% 14% 11%

ipsilateral ③ 1 9 0 2

10% 90% 0% 22%

Interphone meningiomas total 100 14 33 1

88% 12% 33% 7%

highly exposed➃ 2 18 0 0

10% 90% 0% 0%

gliomas total 25 5 9 1

83% 17% 36% 20%

highly exposed➃ 0 21 0 4

0% 100% 0% 19%

-----------------------------------------------------------------------------------------------------------------------

- 95%CI superior limit < 1 for OR<1, and 95%CI inferior limit > 1 for OR>1

② Gliomas, meningiomas, acoustic neuromas, salivary gland tumours

③ only ≥10 year latency

➃ only cases with "highest cumulative call times"
